# Thioredoxin 1 in Prostate Tissue Is Associated with Gleason Score, Erythrocyte Antioxidant Enzyme Activity, and Dietary Antioxidants

**DOI:** 10.1155/2015/728046

**Published:** 2015-08-18

**Authors:** Terrence M. Vance, Gissou Azabdaftari, Elena A. Pop, Sang Gil Lee, L. Joseph Su, Elizabeth T. H. Fontham, Jeannette T. Bensen, Susan E. Steck, Lenore Arab, James L. Mohler, Ming-Hui Chen, Sung I. Koo, Ock K. Chun

**Affiliations:** ^1^Department of Nutritional Sciences, University of Connecticut, Storrs, CT 06269, USA; ^2^Roswell Park Cancer Institute, Buffalo, NY 14263, USA; ^3^Epidemiology and Genomics Research Program, Division of Cancer Control and Population Sciences, National Cancer Institute, Bethesda, MD 20892, USA; ^4^School of Public Health, Louisiana State University Health Sciences Center, New Orleans, LA 70112, USA; ^5^School of Public Health, University of North Carolina at Chapel Hill, Chapel Hill, NC 27599, USA; ^6^Department of Epidemiology and Biostatistics, Cancer Prevention and Control Program, University of South Carolina, Columbia, SC 29208, USA; ^7^David Geffen School of Medicine, University of California, Los Angeles, CA 90095, USA; ^8^Department of Statistics, University of Connecticut, Storrs, CT 06269, USA

## Abstract

*Background*. Prostate cancer is the most common noncutaneous cancer and second leading cause of cancer-related mortality in men in the US. Growing evidence suggests that oxidative stress is involved in prostate cancer.* Methods*. In this study, thioredoxin 1 (Trx 1), an enzyme and subcellular indicator of redox status, was measured in prostate biopsy tissue from 55 men from the North Carolina-Louisiana Prostate Cancer Project. A pathologist blindly scored levels of Trx 1. The association between Trx 1 and the Gleason score, erythrocyte antioxidant enzyme activity, and dietary antioxidant intake was determined using Fisher's exact test.* Results*. Trx 1 levels in benign prostate tissue in men with incident prostate cancer were positively associated with the Gleason score (*P* = 0.01) and inversely associated with dietary antioxidant intake (*P* = 0.03). In prostate cancer tissue, Trx 1 levels were associated with erythrocyte glutathione peroxidase activity (*P* = 0.01). No association was found for other erythrocyte enzymes. Greater Gleason score of malignant tissue corresponds to a greater difference in Trx 1 levels between malignant and benign tissue (*P* = 0.04).* Conclusion*. These results suggest that the redox status of prostate tissue is associated with prostate cancer grade and both endogenous and exogenous antioxidants.

## 1. Introduction

Prostate cancer is the most common noncutaneous cancer and second cause of cancer-related mortality in men in the US, with estimated 238,590 new cases and 29,720 deaths in 2013 [1]. The specific causes of prostate cancer have not yet been determined, but several risk factors have been identified for the disease, which include family history, age, and race [2, 3]. However, the mechanisms by which these and other risk factors, such as lifestyle, contribute to prostate cancer are not clear, and there is likely a great degree of heterogeneity in the causes of the disease.

At the cellular level, a growing body of evidence indicates that oxidative stress is involved in prostate carcinogenesis [4]. Reactive oxygen species can promote carcinogenesis by causing oxidative damage to DNA and macromolecules within cells, altering signal transduction pathways, and promoting a malignant phenotype. Furthermore, genetic polymorphisms in antioxidant enzymes have been associated with cancer risk [5], and prostate cancer risk may be modified by interactions between antioxidant enzyme genotype and dietary antioxidants [6, 7]. Men diagnosed with prostate cancer have been shown to have greater oxidative stress, lower antioxidant enzyme activity [8], and greater levels of urinary isoprostanes, a biomarker of oxidative stress and lipid peroxidation [9, 10]. Furthermore,* in vitro* experiments have implicated the production of reactive oxygen species in the aggressiveness of prostate cancer [11]. Thus, relatively consistent evidence suggests an association between oxidative stress and prostate cancer.

Oxidative stress is regulated in both benign and malignant cells via several nonenzymatic and enzymatic antioxidant mechanisms [4]. Malignant cells have relatively greater levels of reactive oxygen species and compensatory increases in antioxidant enzymes in order to tolerate increased oxidative stress [12]. The expression of several enzymes involved in oxidative stress and detoxification is repressed in prostate cancer, in particular glutathione S-transferase [13]. Furthermore, men with prostate cancer may be subject to greater oxidative stress and exhibit lower activity of erythrocyte glutathione peroxidase and superoxide dismutase, compared with controls [8]. These differences in activity and expression could indicate greater susceptibility to oxidative damage, potentially influencing the development or progression of cancer. Another antioxidant enzyme that is thought to be involved in prostate carcinogenesis is thioredoxin 1 (Trx 1). Thioredoxin reductase 1 (TrxR1) transfers electrons from NADPH to Trx 1, a process that occurs in most living cells and is essential for maintenance of cellular redox status [14]. Both Trx 1 and TrxR1 are increased in prostate cancer [15, 16], with a greater proportion of Trx 1 in an oxidized state, which may reflect redox imbalance and response to greater levels of oxidative stress. Reduced Trx 1 binds to apoptosis signal-regulating kinase 1 (ASK1) and regulates cell death [17]; thus, redox imbalance may promote cancer cell survival. TrxR1 is a selenoenzyme that can be modified by reaction with electrophilic compounds [14] and interact with several dietary phytochemicals that are considered antioxidants [18].

The role of TrxR1 in maintaining Trx 1 in a reduced state and the emerging role of both of these enzymes in carcinogenesis suggest a relationship between TrxR1 and dietary antioxidants. The evidence for a relationship between dietary antioxidants and prostate cancer is inconsistent in humans [19], which is partly due to the relatively small number of dietary antioxidants studied and their diversity in structure and possible mechanisms of action. However, dietary antioxidants, such as flavonoids, have been shown to reduce oxidative damage [20] and promote DNA repair [21] in prostate cancer cells* in vitro*. Dietary antioxidants could have some effect on prostate cancer, possibly by affecting oxidative stress.

The discrepant findings regarding prostate cancer and dietary antioxidants prompted a study of dietary antioxidant intake using total antioxidant capacity (TAC). TAC represents the cumulative antioxidant capacity of individual antioxidants present in diet and supplements [22]. Greater dietary TAC was recently found to be inversely associated with prostate cancer incidence [23]; however, the relationship between dietary antioxidants and redox status in prostate cancer remains unknown. Furthermore, the relationship between Trx 1 in prostate tissue and the activity of antioxidant enzymes in prostate cancer patients is relatively unknown.

The objectives of this study were to measure Trx 1 in benign and malignant prostate tissue of men diagnosed with incident prostate cancer and determine the association between prostate tissue Trx 1 and Gleason score, dietary antioxidant intake, and blood biomarkers of antioxidant enzyme activity. The hypotheses of the current study were as follows: (1) the level of Trx 1 in malignant prostate tissue would be positively associated with Gleason score, (2) high grade cancer would exhibit a greater difference between the Trx 1 level of benign and malignant tissue, and (3) measures of antioxidants and prostate tissue levels of Trx 1 would be inversely associated.

## 2. Materials and Methods

Data collected by the North Carolina-Louisiana Prostate Cancer Project (PCaP), a population based study of incident prostate cancer [24], was used for this study. Briefly, men between 40 and 79 years of age with a first diagnosis of histologically confirmed adenocarcinoma of the prostate on or after July 1, 2004, were eligible to participate in PCaP. Men had to be able to complete the study interview in English and could not live in an institution or nursing home, be cognitively impaired, be under the influence of alcohol, be severely medicated, or be apparently psychotic. Men must have self-identified as either African American or Black or Caucasian or White (European American), when responding to the question “What is your race?” This project was approved by the institutional review boards at the Department of Defense Prostate Cancer Research Program, the University of North Carolina, and Louisiana State University. The current study also was approved by the institutional review board at the University of Connecticut.

Dietary data were collected using a modified version of the National Cancer Institute Diet History Questionnaire (DHQ) [25]. Data for the flavonoid and proanthocyanidin content of foods were added to the DHQ database using Nutrition Data System for Research, version 2011 (Nutrition Coordinating Center, University of Minnesota, Minneapolis, MN). The ABTS (2,2′-azino-bis(3-ethylbenzothiazoline-6-sulfonic acid)) radical anion scavenging activity assay was used previously to measure the vitamin C equivalent antioxidant capacities of individual antioxidants [26] and values were applied to PCaP diet and supplement data. Total antioxidant capacity (TAC) was calculated from the vitamin C equivalent antioxidant capacity of 42 dietary antioxidants (carotenoids, vitamins C and E, flavonoids, proanthocyanidins, and isoflavones) and 5 antioxidants from dietary supplements (vitamin C, alpha-tocopherol, beta-carotene, lycopene, and lutein and zeaxanthin). The antioxidant capacity of an antioxidant was calculated as the product of its reported intake in grams and its vitamin C equivalent antioxidant capacity, and TAC was calculated as the sum of all individual antioxidant capacities.

Paraffin-embedded prostate tissue biopsy specimens were selected from PCaP research subjects who were untreated at the time of sample collection to provide an approximately equal distribution of subjects by site, race, and aggressiveness level to yield a subsample of 59 tissue sections (5 *μ*m in thickness) for analysis. Sections were deparaffinized, rehydrated under an alcohol gradient, and antigen-retrieved using Reveal Decloaker (Biocare Medical, Concord, CA) for 15 minutes at 110°C in a decloaking chamber (Biocare Medical). Sections were blocked for endogenous peroxidase activity using 3% H_2_O_2_, rinsed with deionized, distilled water for 10 minutes at room temperature, blocked with normal goat serum for 1 hour, and incubated overnight at 4°C with rabbit anti-Trx 1 (1 : 1000; Cell Signaling, Danvers, MA). The next day, sections were incubated for 30 minutes at room temperature with Rabbit SignalStain Boost IHC Reagent; the enzymatic activity was revealed using diaminobenzidine (Sigma-Aldrich, St. Louis, MO), counterstained with hematoxylin (Vector Laboratories, Burlingame, CA), and mounted using a permanent mounting medium. Images of stained sections were collected using a Leica DFC0425C camera mounted on a Leica DMRA2 microscope equipped with automated stage. The optimal titer of the Trx 1 primary antibody was determined by assessing immunostain quality after using different dilutions on prostate cancer tissue control sections acquired from a deidentified research subject. Two sections from the deidentified research subject served as positive control, immunostained with Trx 1 or a negative control, immunostained with Trx 1 blocking peptide (Cell Signaling, Danvers, MA).

Prostate cancer Gleason score of biopsy sections was determined by a genitourinary pathologist (GA). Levels of Trx 1 in benign and malignant tissue were visually scored by a genitourinary pathologist (GA) in a blinded fashion. Slides were scored from 0 to 3, with 0 indicating absence of staining and 1 through 3 indicating greater intensity of staining. The difference in Trx 1 between benign and malignant tissue was determined by subtracting the score in benign tissue from that in malignant tissue.

Peripheral blood samples collected from PCaP research subjects were centrifuged and packed erythrocytes were aliquoted and stored at −80°C until analysis. The activities of erythrocyte glutathione peroxidase, glutathione S-transferase, glutathione reductase, and superoxide dismutase were measured in erythrocyte lysates using commercial kits following manufacturer instructions (Cayman Chemical Company, Ann Arbor, MI). Tertiles for dietary TAC and antioxidant enzyme activity were created using SAS PROC RANK. Associations between Trx 1 levels in prostate cancer tissue and Gleason score, erythrocyte antioxidant enzyme activity, and dietary TAC were determined using Fisher's exact test (*α* = 0.05). All *P* values reported are two sided.

A total of 59 prostate biopsy specimens were randomly selected for analyses. Samples were excluded from analyses for the following reasons: (1) sections that contained malignant tissue were not available for four biopsies, (2) four sections fell off the slides during processing, and (3) blood samples were not available for 11 research subjects. The final number used in analyses was 51 for Gleason score, 45 for erythrocyte antioxidant enzymes, and 55 for dietary TAC. All statistical analyses were performed using SAS software, version 9.3 (SAS Institute Inc., Cary, NC).

## 3. Results

**Figure 1:**
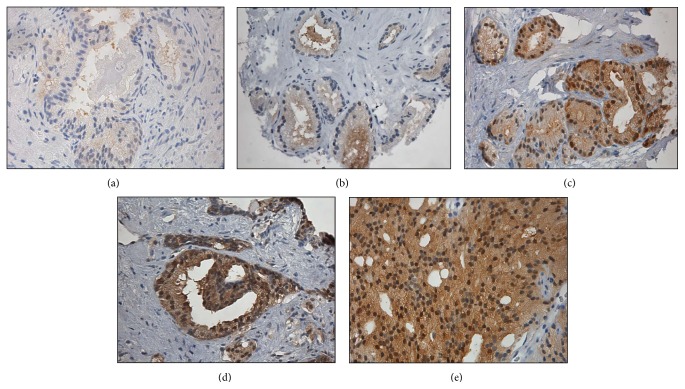
Images of immunohistochemical staining of Trx 1 diagnostic prostate cancer biopsy sections. Sections: (a) benign tissue, (b) Gleason score 3 + 3, (c) Gleason score 3 + 4, (d) Gleason score 4 + 3, and (e) Gleason score 4 + 4.

Descriptive statistics for research subjects are presented in Table 1. The median age of research subjects was 66 years, the median level of prostate-specific antigen was 6.0 ng/dL, and the most common Gleason score was 3 + 4. Images of Trx 1 stained prostate biopsy specimens are shown in Figure 1 for benign tissue and malignant tissue with Gleason scores 3 + 3, 3 + 4, 4 + 3, and 4 + 4.

The associations between Gleason score and Trx 1 are displayed in Table 2. Prostate tissue Trx 1 levels in benign tissue appeared to increase significantly with the Gleason score of malignant tissue. Furthermore, the difference in Trx 1 between benign and malignant tissue was associated significantly with the Gleason score of malignant tissue (*P* = 0.04).

The relationship between prostate tissue Trx 1 and erythrocyte glutathione peroxidase, glutathione reductase, glutathione S-transferase, and superoxide dismutase is shown in Tables 3, 4, 5, and 6, respectively. No association was found between Trx 1 in prostate tissue and activity of erythrocyte glutathione reductase, glutathione S-transferase, or superoxide dismutase. However, Trx 1 levels were lower among research subjects with greater erythrocyte glutathione peroxidase activity (*P* = 0.01).

Dietary TAC, representing a combined dietary antioxidant index, was associated significantly with Trx 1 levels in benign tissue (*P* = 0.03), and greater levels of Trx 1 corresponded to lower dietary TAC (Table 7). There was no significant association between dietary TAC and Trx 1 levels in malignant tissue or the difference in Trx 1 levels between malignant and benign tissue.  Table 8 provides a summary of the associations between Trx 1 and grade, antioxidant enzyme activity, and dietary antioxidant capacity.

## 4. Discussion

Levels of Trx 1 in prostate tissue were found to be associated with Gleason score, antioxidant enzyme activity, and dietary antioxidants. While no association was found between Gleason score and Trx 1 in malignant tissue, a positive association was found with Trx 1 in benign tissue and the difference in Trx 1 level between malignant and benign tissue, indicating that malignant cells have more Trx 1 compared with benign cells and that this difference increases with the Gleason score of malignant tissue (Table 2). While the clinical implications of these findings regarding prostate cancer is not clear, these results may reflect a compensatory response to redox imbalance, since the thioredoxin system is upregulated in prostate cancer [15, 16]. This suggests that more advanced cancer exhibits greater imbalance in redox status.

Glutathione peroxidase activity was associated significantly with Trx 1 level in malignant cells; levels of Trx 1 appeared lower in men with greater glutathione peroxidase activity (Table 3). Since no association was found between Trx 1 and the activity of other erythrocyte antioxidant enzymes, the observed association between Trx 1 and glutathione peroxidase could be due to chance. However, there are several explanations for an absence of effect. Arsova-Sarafinovska et al. [8] found that erythrocyte antioxidant enzyme activity was decreased in prostate cancer patients compared to controls. Since the present study included only men diagnosed with prostate cancer, differences between healthy men and those with prostate cancer may be greater than differences among men with prostate cancer. Other studies have demonstrated that polymorphisms in antioxidant enzymes are related to prostate cancer [5, 6]; antioxidant enzyme activity, as assessed in this study, may not be sensitive enough to capture functional differences in antioxidant enzymes that result from differences in amino acid sequences. Furthermore, erythrocyte antioxidant activity may not reflect prostate tissue antioxidant activity.

Dietary TAC was used to represent antioxidant intake in the diet and was found to be associated significantly with the level of Trx 1 in benign prostate tissue (*P* = 0.03). Benign prostate tissue Trx 1 appears to be inversely associated with dietary TAC, with greater Trx 1 levels associated with lower dietary TAC (Table 7). Much of the evidence regarding dietary antioxidants and prostate cancer is inconsistent, but some dietary antioxidants may be beneficial for the chemoprevention of prostate cancer [19], although little evidence supports an effect of diet on prostate cancer progression. A summary of the associations observed in this study is presented in Table 8.

The primary strength of this study was use of Trx 1 to assess prostate tissue redox status. Trx 1 may indicate redox imbalance in prostate cancer [16]. Expression of TrxR1, the enzyme responsible for maintaining Trx 1 in a reduced form, is increased in castration-resistant prostate cancer [15]. Relatively few studies have been conducted on the thioredoxin system and prostate cancer, and while this system appears to play an important role in prostate cancer, the extent to which this system affects prostate cancer development and progression in humans remains uncertain. To the authors' knowledge, this is the first paper to demonstrate that the redox status of prostate tissue is associated with enzymatic and dietary antioxidants.

This study also has several limitations. The cross-sectional design of the study prohibits establishing a cause and effect relationship between prostate tissue Trx 1 and dietary and erythrocyte antioxidants. Dietary data were collected after diagnosis and knowledge of disease may have biased responses by research subjects, though this would likely have been nondifferential. The number of research subjects included in the study is relatively few, especially those with high Gleason score and Trx 1 levels, which may have contributed to some of the null results.

## 5. Conclusions

This study suggests that the redox status within the prostate is associated with endogenous and exogenous antioxidants. The results of this study and others warrant additional research in humans on the mechanisms underlying the relationship between prostate tissue redox status and carcinogenesis and determining whether the redox status within prostate tissue may influence prostate cancer aggressiveness.

## Figures and Tables

**Table 1:** Descriptive statistics of research subjects.

Characteristic	Median	IQR
Age (years)	66	(61, 71)
PSA (ng/dL)	6.00	(4.7, 11.4)
TAC (mg VCE/d)	576.8	(312.9, 926.4)
GPx (nmol/min/mL)	815	(455.9, 1044.2)
GR (nmol/min/mL)	205.8	(151.8, 305.6)
GST (nmol/min/mL)	49.7	(32.6, 65.4)
SOD (units/mL)^1^	75.5	(68.1, 80)
Gleason score	*N*	%
3 + 3	12	20.3
3 + 4	24	40.7
4 + 3	12	20.3
4 + 4	2	3.4
4 + 5	1	1.7
Family history^2^	12	25.5
BMI^3^		
Underweight	1	1.8
Normal	10	18.2
Overweight	19	34.6
Obese	21	38.2
Morbidly obese	4	7.3
Race		
African American	28	50.9
European American	27	49.1

IQR: interquartile range; BMI: body mass index; PSA: prostate specific antigen; TAC: total antioxidant capacity; VCE/d: vitamin C equivalents per day; GPx: glutathione peroxidase; GR: glutathione reductase; GST: glutathione S-transferase; SOD: superoxide dismutase.

^1^One unit of SOD is the amount of enzyme needed to dismutate 50% of superoxide radicals.

^2^Family history defined as at least one first-degree relative diagnosed with prostate cancer.

^3^BMI categories defined as underweight (<18.5 kg/m^2^), normal (18.5 to 24.9 kg/m^2^), overweight (25.0 to 29.9 kg/m^2^), obese (30.0 to 34.9 kg/m^2^), and morbidly obese (>35.0 kg/m^2^).

**Table 2:** Association between prostate tissue Trx 1 and Gleason score.

	Gleason score								
	3 + 3		3 + 4		4 + 3		4 + 4 or 5 + 4		Total
	*n*	%	*n*	%	*n*	%	*n*	%	
Malignant Trx 1									
1	4	(50)	3	(37)	0	(0)	1	(12)	8
2	6	(25)	13	(54)	4	(16)	1	(4)	24
3	2	(10)	8	(42)	8	(42)	1	(5)	19
Fisher's exact test, *P* = 0.09									

Benign Trx 1									
0	0	(0)	0	(0)	2	(66)	1	(33)	3
1	7	(23)	19	(63)	3	(10)	1	(3)	30
2	5	(33)	4	(26)	5	(33)	1	(6)	15
3	0	(0)	1	(33)	2	(66)	0	(0)	3
Fisher's exact test, *P* = 0.01									

ΔTrx 1									
−1	1	(33)	1	(33)	1	(33)	0	(0)	3
0	5	(31)	5	(31)	4	(25)	2	(12)	16
1	6	(30)	12	(60)	2	(10)	0	(0)	20
2	0	(0)	6	(66)	3	(33)	0	(0)	9
3	0	(0)	0	(0)	2	(66)	1	(33)	3
Total	**12**		**24**		**12**		**3**		**51**
Fisher's exact test, *P* = 0.04									

ΔTrx 1: difference in Trx 1 level between malignant and benign prostate cells.

**Table 3:** Association between prostate tissue Trx 1 and erythrocyte glutathione peroxidase activity.

	Erythrocyte glutathione peroxidase (GPx)						
	T1		T2		T3		Total
	*n*	%	*n*	%	*n*	%	
Malignant Trx 1							
0	2	(50)	0	(0)	2	(50)	4
1	1	(14)	5	(71)	1	(14)	7
2	7	(35)	3	(15)	10	(50)	20
3	6	(42)	7	(50)	1	(7)	14
Fisher's exact test, *P* = 0.01							

Benign Trx 1							
0	0	(0)	1	(100)	0	(0)	1
1	11	(39)	9	(32)	8	(28)	28
2	3	(23)	4	(30)	6	(46)	13
3	2	(66)	1	(33)	0	(0)	3
Fisher's exact test, *P* = 0.55							

ΔTrx 1							
−2	1	(50)	0	(0)	1	(50)	2
−1	1	(25)	2	(50)	1	(25)	4
0	4	(28)	4	(28)	6	(42)	14
1	7	(41)	5	(29)	5	(29)	17
2	3	(42)	3	(42)	1	(14)	7
3	0	(0)	1	(100)	0	(0)	1
Total	**16**		**15**		**14**		**45**
Fisher's exact test, *P* = 0.93							

ΔTrx 1: difference in Trx 1 level between malignant and benign prostate cells.

Median values of tertiles of GPx activity are as follows: T1: 356.6 mol/min/mL; T2: 815.0 nmol/min/mL; T3: 1258.2 nmol/min/mL.

**Table 4:** Association between prostate tissue Trx 1 and erythrocyte glutathione reductase (GR) activity.

	Erythrocyte glutathione reductase (GR)						
	T1		T2		T3		Total
	*n*	%	*n*	%	*n*	%	
Malignant Trx 1							
0	2	(50)	1	(25)	1	(25)	4
1	0	(0)	3	(42)	4	(57)	7
2	9	(45)	6	(30)	5	(25)	20
3	3	(21)	6	(42)	5	(35)	14
Fisher's exact test, *P* = 0.33							

Benign Trx 1							
0	0	(0)	0	(0)	1	(100)	1
1	7	(25)	13	(46)	8	(28)	28
2	5	(38)	2	(14)	6	(46)	13
3	2	(66)	1	(33)	0	(0)	3
Fisher's exact test, *P* = 0.17							

ΔTrx 1							
−2	1	(50)	0	(0)	1	(50)	2
−1	1	(25)	2	(50)	1	(25)	4
0	6	(42)	4	(28)	4	(28)	14
1	5	(29)	5	(29)	7	(41)	17
2	1	(14)	5	(71)	1	(14)	7
3	0	(0)	0	(0)	1	(100)	1
Total	**14**		**16**		**15**		**45**
Fisher's exact test, *P* = 0.62							

ΔTrx 1: difference in Trx 1 level between malignant and benign prostate cells.

Median values of tertiles of GR activity are as follows: T1: 137.8 mol/min/mL; T2: 205.8 nmol/min/mL; T3: 383.8 nmol/min/mL.

**Table 5:** Association between prostate tissue Trx 1 and erythrocyte glutathione S-transferase activity.

	Erythrocyte glutathione S-transferase (GST)						
	T1		T2		T3		Total
	*n*	%	*n*	%	*n*	%	
Malignant Trx 1							
0	2	(50)	1	(25)	1	(25)	4
1	4	(57)	0	(0)	3	(42)	7
2	4	(20)	10	(50)	6	(30)	20
3	6	(42)	5	(35)	3	(21)	14
Fisher's exact test, *P* = 0.21							

Benign Trx 1							
0	0	(0)	1	(100)	0	(0)	1
1	8	(28)	11	(39)	9	(32)	28
2	7	(53)	3	(23)	3	(23)	13
3	1	(33)	1	(33)	1	(33)	3
Fisher's exact test, *P* = 0.65							

ΔTrx 1							
−2	1	(50)	1	(50)	0	(0)	2
−1	2	(50)	1	(25)	1	(25)	4
0	6	(42)	2	(14)	6	(42)	14
1	5	(29)	7	(41)	5	(29)	17
2	2	(28)	4	(57)	1	(14)	7
3	0	(0)	1	(100)	0	(0)	1
Total	**16**		**16**		**13**		**45**
Fisher's exact test, *P* = 0.65							

ΔTrx 1: difference in Trx 1 level between malignant and benign prostate cells.

Median values of tertiles of GST activity are as follows: T1: 19.9 nmol/min/mL; T2: 49.7 nmol/min/mL; T3: 84.4 nmol/min/mL.

**Table 6:** Association between prostate tissue Trx 1 and erythrocyte superoxide dismutase activity.

	Erythrocyte superoxide dismutase (SOD)						
	T1		T2		T3		Total
	*n*	%	*n*	%	*n*	%	
Malignant Trx 1							
0	2	(50)	1	(25)	1	(25)	4
1	1	(14)	3	(42)	3	(42)	7
2	5	(25)	7	(35)	8	(40)	20
3	7	(50)	5	(35)	2	(14)	14
Fisher's exact test, *P* = 0.53							

Benign Trx 1							
0	0	(0)	1	(100)	0	(0)	1
1	11	(39)	8	(28)	9	(32)	28
2	2	(15)	6	(46)	5	(38)	13
3	2	(66)	1	(33)	0	(0)	3
Fisher's exact test, *P* = 0.36							

ΔTrx 1							
−2	1	(50)	0	(0)	1	(50)	2
−1	1	(25)	2	(50)	1	(25)	4
0	3	(21)	6	(42)	5	(35)	14
1	6	(35)	6	(35)	5	(29)	17
2	4	(57)	1	(14)	2	(28)	7
3	0	(0)	1	(100)	0	(0)	1
Total	**16**		**15**		**14**		**45**
Fisher's exact test, *P* = 0.85							

ΔTrx 1: difference in Trx 1 level between malignant and benign prostate cells.

Median values of tertiles of SOD activity are as follows: T1: 66.3 units/mL; T2: 75.9 units/mL; T3: 80.8 units/mL.

**Table 7:** Association between prostate tissue Trx 1 level and dietary total antioxidant capacity.

	Dietary total antioxidant capacity (TAC)						
	T1		T2		T3		Total
	*n*	%	*n*	%	*n*	%	
Malignant Trx 1							
0	0	(0)	2	(50)	2	(50)	4
1	4	(50)	3	(38)	1	(13)	8
2	9	(38)	7	(29)	8	(33)	24
3	5	(26)	7	(37)	7	(37)	19
Fisher's exact test, *P* = 0.64							

Benign Trx 1							
0	0	(0)	3	(100)	0	(0)	3
1	12	(38)	11	(34)	9	(28)	32
2	3	(18)	5	(29)	9	(53)	17
3	3	(100)	0	(0)	0	(0)	3
Fisher's exact test, *P* = 0.03							

ΔTrx 1							
−2	0	(0)	1	(50)	1	(0)	2
−1	1	(20)	2	(40)	2	(40)	5
0	7	(44)	4	(25)	5	(32)	16
1	9	(45)	6	(30)	5	(25)	20
2	1	(11)	3	(33)	5	(56)	9
3	0	(0)	3	(100)	0	(0)	3
Total	**18**		**19**		**18**		**55**
Fisher's exact test, *P* = 0.34							

ΔTrx 1: difference in Trx 1 level between malignant and benign prostate cells.

Median values of tertiles of dietary TAC are as follows: T1: 251.2 mg VCE/d; T2: 576.8 mg VCE/d; T3: 1181.0 mg VCE/d.

**Table 8:** Summary of associations between prostate cancer Gleason score, antioxidant enzymes, and dietary total antioxidant capacity and Trx 1 levels in prostate tissue based on results of the present study: (+) indicates a positive association and (−) a negative association; empty cells indicate no evidence of an association.

	Trx 1	
	Malignant tissue	Benign tissue
Gleason score	+	+
GPx	−	
GR		
GST		
SOD		
TAC		−

GPx: erythrocyte glutathione peroxidase activity; GR: erythrocyte glutathione reductase activity; GST: erythrocyte glutathione S-transferase activity; SOD: erythrocyte superoxide dismutase activity; TAC: dietary total antioxidant capacity.

^*∗*^While the association between Gleason score and Trx 1 in malignant tissue was not statistically significant (*P* = 0.09), the data in Table 2 displays evidence of a positive association.

## References

[1] Siegel R., Ma J., Zou Z., Jemal A. (2014). Cancer statistics, 2014. *CA Cancer Journal for Clinicians*.

[2] Brawley O. W. (2012). Prostate cancer epidemiology in the United States. *World Journal of Urology*.

[3] Patel A. R., Klein E. A. (2009). Risk factors for prostate cancer. *Nature Clinical Practice Urology*.

[4] Paschos A., Pandya R., Duivenvoorden W. C. M., Pinthus J. H. (2013). Oxidative stress in prostate cancer: changing research concepts towards a novel paradigm for prevention and therapeutics. *Prostate Cancer and Prostatic Diseases*.

[5] Klaunig J. E., Kamendulis L. M., Hocevar B. A. (2010). Oxidative stress and oxidative damage in carcinogenesis. *Toxicologic Pathology*.

[6] Li H., Kantoff P. W., Giovannucci E. (2005). Manganese superoxide dismutase polymorphism, prediagnostic antioxidant status, and risk of clinical significant prostate cancer. *Cancer Research*.

[7] Goodman M., Bostick R. M., Ward K. C. (2006). Lycopene intake and prostate cancer risk: effect modification by plasma antioxidants and the XRCC1 genotype. *Nutrition and Cancer*.

[8] Arsova-Sarafinovska Z., Eken A., Matevska N. (2009). Increased oxidative/nitrosative stress and decreased antioxidant enzyme activities in prostate cancer. *Clinical Biochemistry*.

[9] Brys M., Morel A., Forma E. (2013). Relationship of urinary isoprostanes to prostate cancer occurence. *Molecular and Cellular Biochemistry*.

[10] Barocas D. A., Motley S., Cookson M. S. (2011). Oxidative stress measured by urine F2-isoprostane level is associated with prostate cancer. *Journal of Urology*.

[11] Kumar B., Koul S., Khandrika L., Meacham R. B., Koul H. K. (2008). Oxidative stress is inherent in prostate cancer cells and is required for aggressive phenotype. *Cancer Research*.

[12] Cairns R. A., Harris I. S., Mak T. W. (2011). Regulation of cancer cell metabolism. *Nature Reviews Cancer*.

[13] Singal R., van Wert J., Bashambu M. (2001). Cytosine methylation represses glutathione S-transferase P1 (GSTP1) gene expression in human prostate cancer cells. *Cancer Research*.

[14] Arnér E. S. J., Holmgren A. (2006). The thioredoxin system in cancer. *Seminars in Cancer Biology*.

[15] Singh S. S., Li Y., Ford O. H. (2008). Thioredoxin reductase 1 expression and castration-recurrent growth of prostate cancer. *Translational Oncology*.

[16] Shan W., Zhong W., Zhao R., Oberley T. D. (2010). Thioredoxin 1 as a subcellular biomarker of redox imbalance in human prostate cancer progression. *Free Radical Biology & Medicine*.

[17] Holmgren A., Lu J. (2010). Thioredoxin and thioredoxin reductase: current research with special reference to human disease. *Biochemical and Biophysical Research Communications*.

[18] Arnér E. S. J. (2009). Focus on mammalian thioredoxin reductases—important selenoproteins with versatile functions. *Biochimica et Biophysica Acta*.

[19] Vance T. M., Su J., Fontham E. T. H., Koo S. I., Chun O. K. (2013). Dietary antioxidants and prostate cancer: a review. *Nutrition and Cancer*.

[20] Sharma H., Kanwal R., Bhaskaran N., Gupta S. (2014). Plant flavone apigenin binds to nucleic acid bases and reduces oxidative DNA damage in prostate epithelial cells. *PLoS ONE*.

[21] Gao K., Henning S. M., Niu Y. (2006). The citrus flavonoid naringenin stimulates DNA repair in prostate cancer cells. *Journal of Nutritional Biochemistry*.

[22] Serafini M., Del Rio D. (2004). Understanding the association between dietary antioxidants, redox status and disease: is the Total Antioxidant Capacity the right tool?. *Redox Report*.

[23] Russnes K. M., Wilson K. M., Epstein M. M. (2014). Total antioxidant intake in relation to prostate cancer incidence in the health professionals follow-up study. *International Journal of Cancer*.

[24] Schroeder J. C., Bensen J. T., Su L. J. (2006). The North Carolina-Louisiana Prostate Cancer Project (PCaP): methods and design of a multidisciplinary population-based cohort study of racial differences in prostate cancer outcomes. *Prostate*.

[25] National Institutes of Health ARP (2007). *Diet History Questionnaire, Version 1.0*.

[26] Floegel A., Kim D.-O., Chung S.-J. (2010). Development and validation of an algorithm to establish a total antioxidant capacity database of the US diet. *International Journal of Food Sciences and Nutrition*.

